# Supplementary material for: The Diagnostic Performance of a Clinical Diagnosis of Diabetic Kidney Disease
Source: Life (Basel). 2023 Jun 30;13(7):1492. doi: 10.3390/life13071492 (PMC10381424; doi:10.3390/life13071492)
Supplement: Supplementary file 1 [file life-13-01492-s001.zip › life-2432809-supplementary.pdf]

Supplementary Table S1: Listed indications for biopsy and biopsy diagnoses at individual patient level.

Patient	Gender	Age (y)	DM Treatment	eGFR	Proteinuria category	RAAS blocker therapy	Clinical diagnosis includes DKD?	Indication for biopsy	DKD present on biopsy	Other diagnoses present on biopsy
1	F	74.7	I	33	4	Y	Y	11g proteinuria/24h. Negative vasculitis screen. Coexisting membranous nephropathy?	Y	Nil
2	M	49.8	I	29	3	Y	Y	More rapid than expected decline in GFR. Microscopic haematuria. Coexisting GN?	Y	Nil
3	F	60.4	I	17	2	Y	Y	Rapidly decreasing renal function in a diabetic with microalbuminuria only.	Y	Nil
4	M	70.3	I	42	4	Y	Y	Nephrotic syndrome.	Y	Nil
5	F	61.8	I	61	3	Y	Y	Worsening proteinuria and kidney function in patient with DM 1.	Y	Nil
6	M	31.0	I	14	4	Y	Y	Nephrotic syndrome.	Y	Nil
7	M	43.4	I	47	4	Y	Y	HT and DM. Microscopic haematuria. Nephrotic proteinuria.	Y	Nil
8	M	51.6	O	38	3	Y	Y	Worsening proteinuria. Microscopic haematuria. Only DKD present?	Y	Nil
9	M	64.6	I	31	3	Y	Y	Worsening proteinuria. Worsening kidney function. Only DKD present?	Y	Nil
10	F	27.4	I	31	4	Y	Y	Nephrotic proteinuria. Microscopic haematuria.	Y	Nil

11	M	59.2	I	41	4	Y	Y	Worsening kidney function in diabetic with proteinuria and also microscopic haematuria.	Y	Nil
12	F	41.5	I	45	4	Y	Y	Severely nephrotic with >10g proteinuria/24h.<br>Type 1 DM.<br>Low titre ANA.<br>Only DKD?	Y	Nil
13	M	65.7	D	50	1	Y	Y	Development of new nephrotic syndrome with microscopic haematuria.	Y	Nil
14	M	51.8	I	8	4	Y	Y	Rapid decline in kidney function in diabetic with nephrotic proteinuria.<br>Exclude superimposed GN.	Y	Nil
15	F	47.1	I	30	4	Y	Y	Recent onset nephrotic syndrome.<br>Rapid decline in kidney function.<br>Exclude superimposed GN.	Y	Nil
16	M	52.0	O	103	3	Y	Y	DM only recently diagnosed.<br>Proteinuria and kidney function worse than expected.<br>Exclude other pathology.	Y	Nil
17	F	64.9	I	28	4	Y	Y	13g proteinuria/24h.<br>Exclude co-existing membranous nephropathy.	Y	Nil
18	F	58.2	O	25	4	Y	Y	DM2 with nephrotic proteinuria.<br>Obese.<br>Exclude co-existing FSGS.	Y	Nil
19	M	67.7	I	22	4	Y	Y	Nephrotic syndrome.	Y	Nil

								Microscopic haematuria.<br>Positive ANA (negative dsDNA and ENA, normal Complement)<br>Exclude co-existing GN.		
20	M	61.5	I	37	3	Y	Y	Recent onset nephrotic range proteinuria despite good DM control.	Y	Nil
21	F	58.4	I	29	4	Y	Y	Nephrotic syndrome.<br>Impaired kidney function.<br>Microscopic haematuria.<br>Positive ANA (1:160).	Y	Nil
22	F	49.6	I	19	4	Y	Y	DM 1.<br>Nephrotic proteinuria.<br>Impaired kidney function.<br>Microscopic haematuria.<br>Exclude co-existing GN.	Y	Nil
23	F	48.4	I	77	4	Y	Y	DM2.<br>Hypopituitarism.<br>Nephrotic range proteinuria.<br>Obese.<br>Superimposed FSGS?	Y	Nil
24	F	75.9	O	20	3	Y	Y	Worsening kidney function.<br>Worsening proteinuria.<br>Only DKD?	Y	Nil
25	F	50.7	O	31	4	Y	Y	DM2 with family history.<br>Nephrotic range proteinuria.<br>Microscopic haematuria.<br>Positive MPO-ANCA.<br>DKD or microscopic polyangiitis?	Y	Microscopic polyangiitis.
26	M	59.7	I	55	4	Y	Y	DM2.<br>Nephrotic proteinuria.	Y	FSGS (secondary).

								Microscopic haematuria.		
27	M	61.4	O	40	4	Y	Y	Poorly controlled DM2.<br>Nephrotic proteinuria.<br>Low level serum paraprotein.<br>Normal serum free light chain ratio.<br>No Bence Jones protein.	Y	FSGS (secondary).
28	M	56.7	I	35	4	Y	Y	DM2.<br>Rheumatoid arthritis.<br>Nephrotic range proteinuria.<br>Impaired kidney function.<br>Superimposed amyloid?	Y	FSGS (secondary).
29	F	52.2	O	101	4	Y	Y	Nephrotic range proteinuria.<br>Microscopic haematuria.<br>Co-existing GN?	Y	IgA nephropathy.
30	M	55.7	O	69	3	Y	N	Non-nephrotic proteinuria.<br>Microscopic haematuria.<br>GN more likely?	Y	Light chain deposition disease.
31	F	43.5	I	64	4	Y	Y	SLE with low complement and raised dsDNA.<br>Newly positive lupus anticoagulant.<br>Grade of lupus nephritis?	Y	Lupus nephritis (ISN/RPN Class III)
32	F	67.5	O	45	3	Y	Y	DM2 but also Sjogren's.<br>Recent worsening of kidney function.<br>Any involvement from Sjogren's?	Y	Sjogren's syndrome.
33	F	48.8	I	55	3	Y	Y	DM1.<br>Hepatitis C.<br>Proteinuria.<br>Microscopic haematuria.<br>Co-existing MPGN?	Y	FSGS (secondary).

34	M	51.3	I	27	4	Y	Y	Proteinuria.<br>Impaired kidney function.<br>Microscopic haematuria.<br>Co-existing GN?	Y	MCGN (MPGN) type 1.
35	F	40.7	I	30	4	Y	Y	Poorly controlled DM1.<br>Nephrotic proteinuria.	Y	FSGS (secondary).
36	M	58.5	O	101	3	Y	Y	DM2.<br>Hypertension.<br>Proteinuria.<br>Normal kidney function.	Y	HT.
37	F	55.2	I	9	2	N	Y	Acute deterioration in kidney function.<br>DM2 (>5y).<br>Obese.<br>Microalbuminuria only.<br>Raised MPO-ANCA.<br>Microscopic polyangiitis only?	Y	ANCA associated vasculitis (Microscopic polyangiitis).
38	F	48.1	I	34	4	Y	Y	DM2.<br>Nephrotic proteinuria.<br>No other micro- or macro-vascular complications.	Y	IgA nephropathy.
39	F	61.9	I	39	4	Y	Y	DM2.<br>Obese.<br>Proteinuria.<br>Impaired kidney function.<br>Anaemic.	N	IgA nephropathy.
40	M	76.0	D	31	2	N	Y	Acute on chronic kidney disease.<br>DM2 on diet only.<br>Minimal proteinuria.	N	Ischaemic nephrosclerosis.
41	F	70.8	O	50	3	Y	Y	Acute decline in function.<br>DM2.	N	Acute tubular necrosis (ATN)

								Proteinuria.		
42	F	47.6	O	97	3	Y	N	Proteinuria <1g/day.<br>Strong positive ANA but dsDNA and ENA negative,<br>Complement normal.	N	FSGS
43	F	73.5	O	90	3	Y	N	Proteinuria >2g/day.<br>Microscopic haematuria.	N	MCGN (MPGN) type unspecified.
44	F	65.1	D	42	3	Y	Y	AKI.<br>Recent antibiotic therapy (Co-Amoxyclav).<br>Interstitial nephritis?	N	ATN.
45	F	78.3	O	41	1	Y	Y	AKI but slow to recover.<br>Eosinophils in urine.<br>Interstitial nephritis?	N	ATN.
46	M	59.8	I	90	3	Y	N	Proteinuria.<br>Microscopic haematuria.<br>Negative vasculitis screen.	N	FSGS.
47	M	46.4	O	57	3	Y	N	Proteinuria.<br>CKD 3.<br>Family history of kidney failure.	N	IgA nephropathy.
48	M	73.0	I	38	2	Y	N	AKI and acute liver injury following IV antibiotic therapy.<br>Widespread maculopapular rash.<br>Eosinophilia.<br>Interstitial nephritis?	N	Acute interstitial nephritis.
49	F	63.3	O	35	3	Y	N	Myeloma, any renal involvement?	N	Myeloma.
50	M	73.7	I	20	4	Y	N	Rapidly deteriorating kidney function.<br>Nephrotic proteinuria.	N	FSGS (Secondary).

								New microscopic haematuria.<br>GN?		
51	M	54.3	I	24	3	N	N	Rapidly deteriorating kidney function.<br>High anti-streptolysin O titre despite no preceding streptococcal infection	N	Anti GBM disease.
52	M	65.8	O	91	3	Y	Y	DM.<br>Hypertension.<br>Obese.<br>Proteinuria.<br>DKD v FSGS?	N	Hypertensive nephrosclerosis.<br>FSGS.
53	M	70.5	O	43	1	Y	N	CKD.<br>Known CLL.<br>Any kidney involvement?	N	Ischaemic nephrosclerosis.<br>No CLL involvement.
54	M	74.2	D	40	1	Y	N	Worsening kidney function.<br>No proteinuria.<br>Known urothelial cancer.<br>Cause for decline?	N	HT.

AKI acute kidney injury, ANA antinuclear antibodies, ATN acute tubular necrosis, CKD chronic kidney disease, CLL chronic lymphocytic leukaemia, DKD diabetic kidney disease, DM (1 or 2) diabetes mellitus (types 1 or 2), dsDNA double stranded DNA antibodies, ENA extractable nuclear antigen antibodies, FSGS focal segmental glomerulosclerosis, HT hypertension, MCGN mesangiocapillary GN (MPGN membranoproliferative GN)
